# Trends in Childhood Behavioral, Mental, and Developmental Problems (2019–2022) Using the National Survey of Children’s Health

**DOI:** 10.3390/pediatric16040084

**Published:** 2024-11-11

**Authors:** Austin Lent, Ayden Dunn, Nada Eldawy, Vama Jhumkhawala, Meera Rao, Joshua Sohmer, Lea Sacca

**Affiliations:** Department of Population Health and Social Medicine, Charles E. Schmidt College of Medicine, Florida Atlantic University, Boca Raton, FL 33431, USA; alent2018@health.fau.edu (A.L.); adunn2023@health.fau.edu (A.D.); neldawy2022@health.fau.edu (N.E.); vjhumkhawala2022@health.fau.edu (V.J.); mrao2022@health.fau.edu (M.R.); jsohmer2022@health.fau.edu (J.S.)

**Keywords:** depression, anxiety, childhood developmental problems, caregiver emotional health, social determinants of health

## Abstract

Background: This study examines nationwide prevalence of US childhood behavioral, mental, and developmental problems in the 6–11 age group category between 2019 and 2022, and several key metrics related to caregiver social determinants of health. Methods: We used NSCH data for 6–11-year-old children for the years 2019–2022. Summary statistics for the selected sample were generated and binary logistic regressions were conducted for each severity level binary variable for each study year with caregiver mental or emotional health as a covariate. Results: Our study found significant associations between severe childhood anxiety and “fair” or “poor” caregiver rankings of mental and emotional health for both the 2020 and 2022 survey years. Our study also found that caregiver mental and emotional health may play a role in childhood ADD and ADHD prevalence rates as caregivers who ranked their mental health as “fair” or “good” had significantly higher odds of having a child with severe ADD or ADHD than caregivers who reported “excellent” mental and emotional health. Conclusion: These findings support prospects of an increase in the need for developmental health services, thus necessitating efforts towards effective allocation and focus of developmental treatment programs tailored to children and pediatric population groups.

## 1. Introduction

Childhood mental, behavioral, and developmental disorders (MBDDs) represent a range of problems that can significantly affect the psychological and social well-being of children [1]. These conditions can present starting in preschool and include anxiety disorders, autism spectrum disorders (ASD), intellectual disabilities, conduct disorders, attention-deficit/hyperactivity disorder (ADHD), and depressive disorders [1,2,3]. Other major childhood disorders include other types of disruptive behavioral problems, such as temper tantrums, and oppositional or defiant disorders [3]. In the United States, nearly one-fifth of children and young people aged 3–17 have an MBDD [4]. Among U.S. high school students, suicidal behaviors increased more than 40% in the decade preceding 2019 [4]. In fact, mental health challenges comprise the leading cause of death and disability in individuals aged 3–17 [4]. Tkacz et al. [5] found a 29.4% increase in incidence and a 34.6% increase in the prevalence of mental health disorders, most notably for anxiety, depression, and eating disorders, based on health insurance claims for children aged 4–17 between 2012 and 2018. The increase in diagnosis rates and diagnosis occurring at earlier ages is attributable to multiple causes, including decreased stigma towards identifying such illnesses in younger populations, the social impacts of the COVID-19 pandemic, and increased effects of social media through cyberbullying and the promotion of an extrinsic, validation-seeking mindset [5]. The rates observed across the general pediatric population also hold for younger children. In 2016, 17.4% of children in the U.S. aged 2–8 had a diagnosed MBDD [6]. Highlighting the impact of COVID-19 on this population, mental health-related visits for children aged 5–11 years in the U.S. increased 24% from weeks 12 through 42 of 2019 to the same period of 2020 [7].

Several risk factors contribute to the development of early onset childhood MBDDs. Adverse childhood events (ACEs) are common predisposing factors for the onset of such problems early on in a child’s life and later with presentation in adolescence. Familial dysfunction, which includes household dysfunction, divorce, criminality, parental mental illness, or jobless parents for more than 9 months, is a significant source of ACEs [8]. Several of these adversities are more commonly experienced in families of lower socioeconomic status, underscoring the impact of social determinants in the early development of such disorders. This is illustrated by Cree et al.’s [6] finding that 22% of children in the lowest income threshold (<100% of the federal poverty line) have been diagnosed with an MBDD compared to 13.9% of children in the highest income threshold (>400% of the federal poverty line). Studies have also illustrated that the age of exposure to adversity can impact when a child develops a mental health problem [9]. For example, since early ages are critical for child development, ACEs faced earlier in life, such as postpartum maternal depression, can affect a child’s ability to form relationships, which contributes to the early development of an MBDD [9]. Similarly, prolonged exposure duration and increased exposure intensity have been associated with MBDD development [9].

Multiple studies [10,11,12,13] have shown that the well-being of primary caregivers influences early childhood development. Parental mental illness can impact the cognitive and behavioral development of children by hindering the parent–child relationship [12]. For instance, parental depression is associated with decreased sensitivity towards children, accuracy in interpreting children’s emotions, and involvement in the child’s life combined with increased withdrawal or intrusion [12]. Additionally, parental caregiving is an important factor that influences how a child interacts with the environment, a vital aspect of childhood development [10]. The presence of challenging social circumstances, such as poverty and lack of safety, can increase family stress, leading to the emergence of caregiver mental health issues and subsequently impacting the quality of care that they are able to provide their children [11]. Conversely, caregiver mental and emotional well-being can mediate the relationship between ACEs and child internalizing symptoms, which helps explain why not all children with familial dysfunction experience MBDDs [13].

Despite the evidence that presently exists regarding the relationship between caregiver mental health and child well-being, further exploration is necessary. Many of the studies assessing parental mental health focus on postnatal mental health, particularly maternal depression [12]. However, it is also important to understand the impacts of the mental well-being of both parents, particularly as it relates to social determinants of health [13,14]. School-age, approximately 6–11 years old, is the period in which complex emotional development and cognitive development occur, and thus requires additional study to understand the pertinent factors that can influence a child’s environment and their growth [15]. Our study seeks to fill the gap in the literature by providing fresh estimates of change in the nationwide prevalence of US childhood behavioral (behavioral conduct problems), mental (anxiety and depression), and developmental problems (ASD and ADD/ADHD) in the 6–11 age group category between 2019 (baseline year) and 2022, both in terms of the overall burden of childhood behavioral, mental, and developmental problems and caregiver mental and emotional health.

## 2. Methods

### 2.1. Data, Setting, and Population

The National Survey of Children’s Health (NSCH) is a household survey on factors related to the physical and emotional health of children aged 0–17 years old in the United States and has been collected annually since 2016 [16,17] The NSCH is the only survey on the health and well-being of children, families, and their communities at the national and state levels. Households with children are identified by a screener instrument, and one child is randomly selected from each household. The selected child is the subject of a follow-up topical questionnaire, which is then completed by an adult who is familiar with the health of the child. Surveys were offered in English and Spanish and could be completed online, on paper, or over the phone with a telephone questionnaire assistance (TQA) agent. One of three age-based topical questionnaires is provided based on the sample child’s age: 0 through 5 years old, 6 through 11 years old, and 12 through 17 years old. We used NSCH data for 6–11-year-old children for the years 2019 (*n* = 9029), 2020 (*n* = 13,097), 2021 (*n* = 14,007), and 2022 (*n* = 15,334). The overall survey response rate for each study year was: 42.4% (2019), 42.4% (2020), 40.3% (2021), and 39.1% (2022). We decided to focus this study on the 6–11-year-old age range due to evidence from a scoping review of a generalized increase in mental health problems among school-aged children after the COVID-19 pandemic [18]. This paper was deemed exempt from the Florida Atlantic University IRB review because it consists of secondary data analysis from a public database.

### 2.2. Outcome Variables

All our outcome measures were based on survey items that asked parents to report on whether their child has ever received a specific diagnosis for five types of childhood mental illnesses. We generated five variables measuring the parent-reported prevalence of mental health and development difficulties for children aged 6–11 years old Responses for each of the five variables were collected based on the three questions asked on mental health and development difficulties for children aged 6–11 years old ((1) ever being diagnosed with anxiety, depression, autism/ASD, ADD/ADHD, or behavioral conduct problems; (2) current diagnosis of child with condition; and (3) severity of the condition). Survey items consisted of “Has a doctor or other health care provider EVER told you that this child has...” for anxiety, depression, autism/ASD, or ADD/ADHD or “Has a doctor, other health care provider, or educator EVER told you that this child has...” for behavioral/conduct problems. Subsequently, the survey asks, “If yes, does this child CURRENTLY have the condition?” for all 5 variables. Lastly, the survey asks, “If yes, is it: mild, moderate, or severe?” for all 5 variables.

### 2.3. Control Variables

Multiple studies [19,20,21] have identified an association of parental mental health with child mental health in the United States, including during the COVID-19 pandemic. Thus, we considered the mental and emotional health of caregiver as the control variable based on the survey question, “In general, how is your mental or emotional health?” The answer options were: “excellent”, “very good”, “good”, “fair”, or “poor”. This item was the only survey item used to assess caregiver mental and emotional health.

### 2.4. Analysis

Data analysis was carried out with IBM SPSS Statistics (version 29). First, we selected responses for children between 6 and 11 years old, inclusive. For the outcome variables, the answers were coded as follows: “yes” = 1, “no” = 2, logical skip = 2, all other responses = systems missing. Severity was recoded into a new binary variable: “not severe” = 1 if the parents responded “mild” or “moderate”, and “severe” = 2 if the parents responded “severe.” For the caregiver mental/emotional health covariate, the answers were coded: “excellent” = 1, “very good” = 2, “good” = 3, “fair” = 4, and “poor” = 5.

We generated summary statistics for the selected sample by computing means, standard deviations, frequencies, and proportions for each study year. Figures were then created for the proportion of children currently experiencing each condition over the four study years and for the proportion of children with a severe condition among those currently experiencing that condition over the four study years. We then ran binary logistic regressions for each severity level binary variable for each study year with caregiver mental or emotional health as a covariate (with “excellent” mental or emotional health serving as the reference status).

## 3. Results

### 3.1. Prevalence of MBDDs and Severe MBDDs in Children Aged 6–11 Years Old from 2019–2022

**Figure 1:**
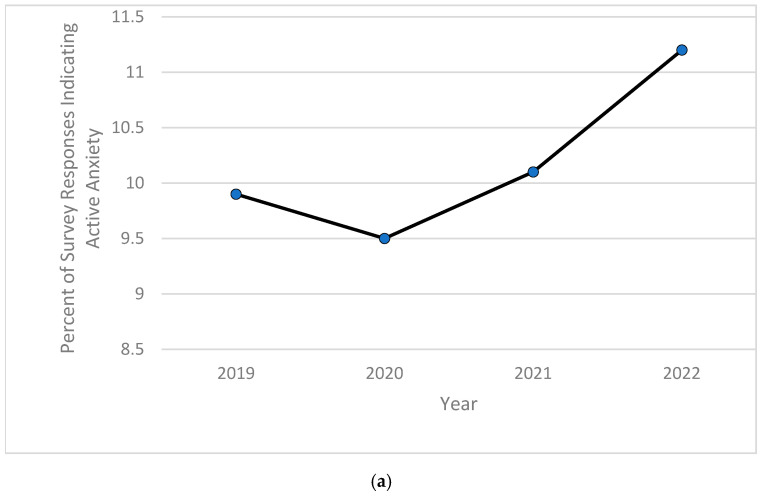

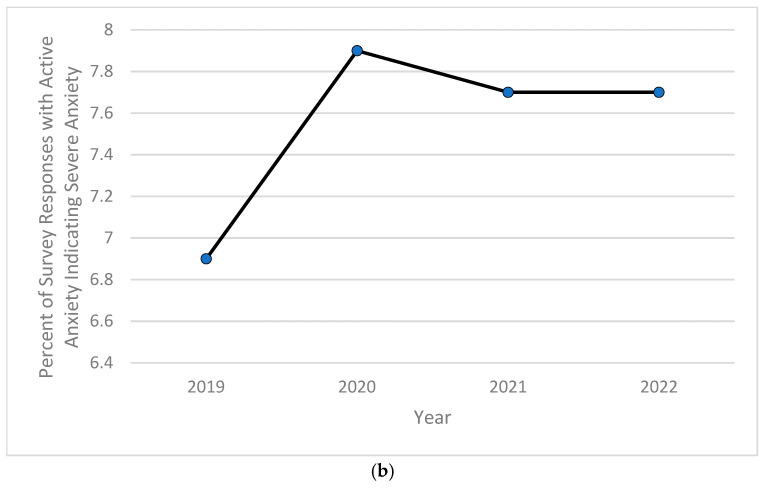
(**a**) Percentage of children 6–11 years old currently experiencing anxiety during study period of 2019–2022; (**b**) percentage of children 6–11 years old experiencing severe anxiety among those currently experiencing anxiety during study period of 2019–2022.

**Figure 2:**
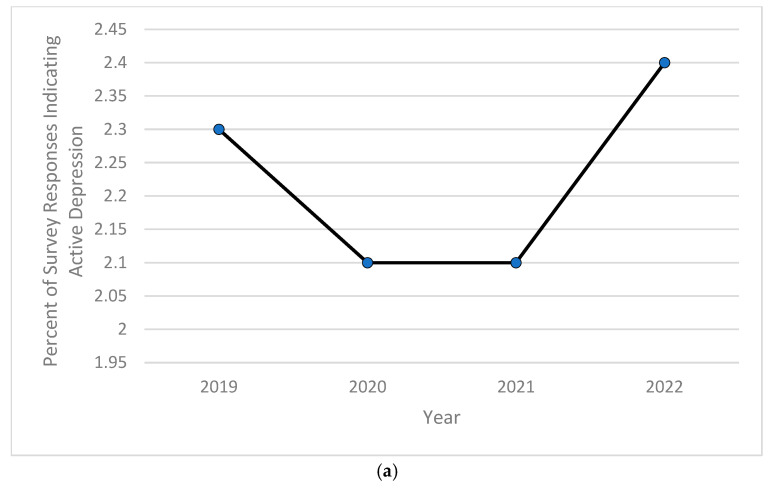

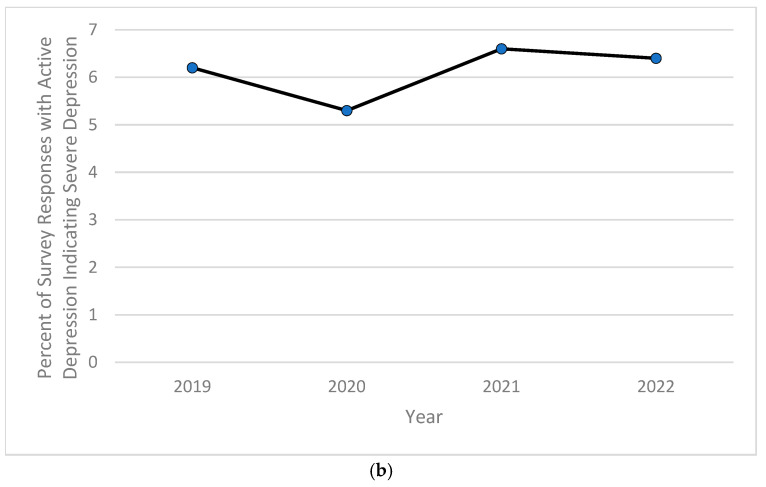
(**a**) Percentage of children 6–11 years old currently experiencing depression during study period of 2019–2022; (**b**) percentage of children 6–11 years old experiencing severe depression among those currently experiencing depression during study period of 2019–2022.

**Figure 3:**
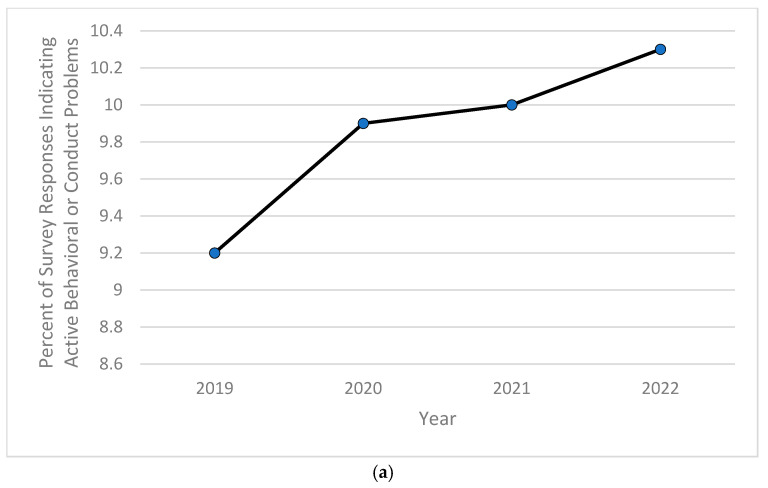

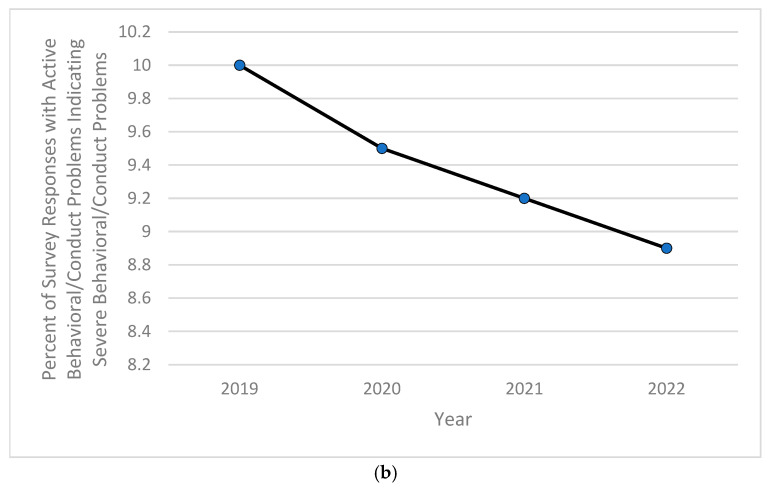
(**a**) Percentage of children 6–11 years old currently experiencing behavioral or conduct problems during study period of 2019–2022; (**b**) percentage of children 6–11 years old experiencing severe behavioral or conduct problems among those currently experiencing behavioral or conduct problems during study period of 2019–2022.

**Figure 4:**
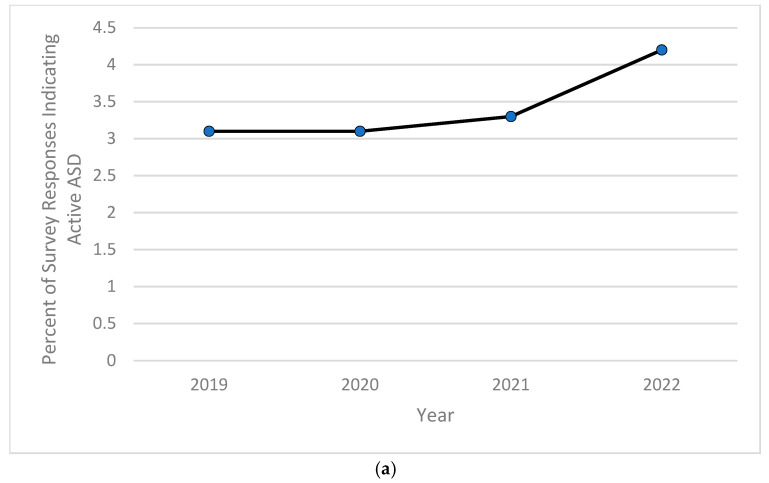

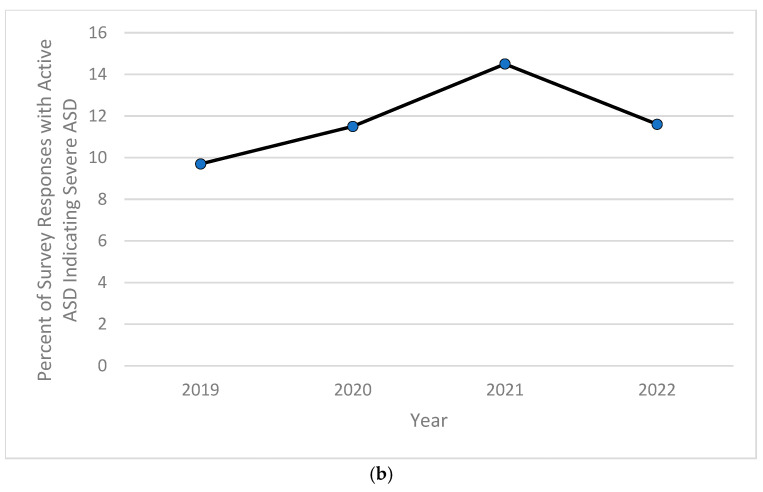
(**a**) Percentage of children 6–11 years old currently experiencing autism or autism spectrum disorder (ASD) during the study period of 2019–2022; (**b**) percentage of children 6–11 years old experiencing autism or autism spectrum disorder (ASD) during the study period of 2019–2022.

**Figure 5:**
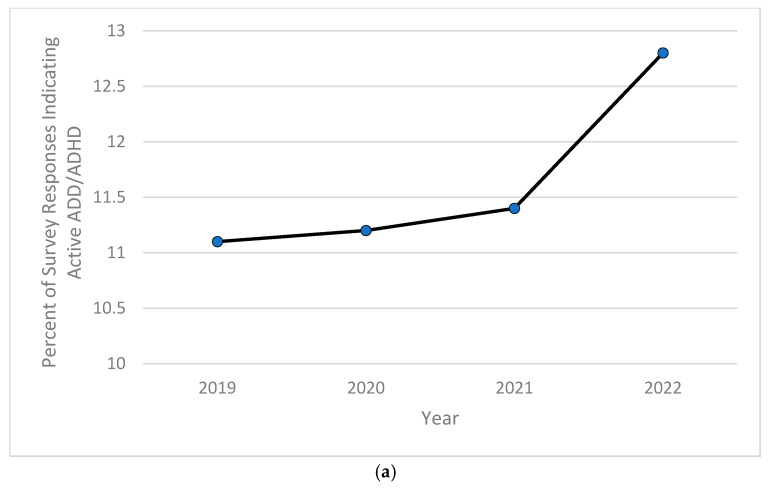

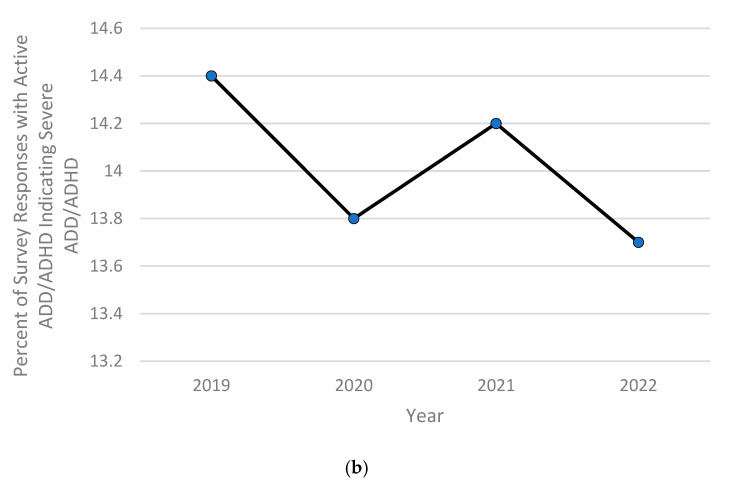
(**a**) Percentage of children 6–11 years old currently experiencing ADD/ADHD; (**b**) percentage of children 6–11 years old experiencing severe ADD/ADHD during the study period of 2019–2022.

Based on the computed descriptive frequencies and proportions, the prevalence of children currently diagnosed with anxiety significantly increased from 9.5% in 2020 to over 11% in 2022 (Figure 1a), while the percentage of children experiencing severe forms of anxiety peaked in 2020 at almost 8%, then decreased and stabilized at around 7.7% in both years 2021 and 2022 (Figure 1b). As for depression, the childhood prevalence of this mental health disorder increased significantly from 2.3% in 2019 and 2.1% in both 2020 and 2021 to 2.4% in 2022 (Figure 2a). The severity of childhood depression fluctuated between 5–7%, reaching a high of 6.8% in 2021 (Figure 2b). A significantly ongoing upward trend in currently diagnosed children with behavioral or conduct problems was observed in the 2019–2022 period, with the prevalence of such disorders increasing from 9.2% in 2019 to 10.3% in 2022 (Figure 3a). However, the severity of childhood behavioral and conduct problems was observed to decrease during the same time period from 10% in 2019 to 8.9% in 2022 (Figure 3b). The prevalence of ASD was relatively stable and around 3% between 2019 and 2020, then steadily increased to 4.25% in 2022 (Figure 4a). However, ASD severity was relatively high across this timeframe, increasing from 9.8% in 2019 to 14.5% in 2021 and decreasing to 11.8% in 2022 (Figure 4b). Finally, the prevalence of ADD/ADHD witnessed an ongoing upward trend in reported cases, reaching its peak of 12.8% in 2022 (Figure 5a). As for the severity of ADD/ADHD, fluctuations were apparent, ranging from 14.4% in 2019 to 13.8% in 2020 and from 14.2% in 2021 to 13.7% in 2022 (Figure 5b).

### 3.2. Regression Analysis

#### 3.2.1. Anxiety

In 2020, caregivers who ranked their mental and emotional health as fair had 2.311 higher odds (*p* = 0.016, 1.166–4.583) of having a child with severe anxiety compared to a caregiver with excellent mental and emotional health. In 2022, caregivers who ranked their mental and emotional health as fair or poor had 2.406 odds (*p* = 0.027, 1.102–5.249) and 5.907 higher odds (*p* < 0.001, 2.282–15.294) of having a child with severe anxiety compared to a caregiver with excellent mental and emotional health, respectively. All other associations were not significant (Table 1).

#### 3.2.2. Behavioral or Conduct Problems

In 2019, caregivers who ranked their mental and emotional health as fair had 2.455 higher odds (*p* = 0.037, 1.057–5.702) of having a child with severe behavioral or conduct problems compared to a caregiver with excellent mental and emotional health. In 2020, caregivers who ranked their mental and emotional health as poor had 3.729 higher odds (*p* = 0.015, 1.297–10.721) of having a child with severe behavioral or conduct problems compared to a caregiver with excellent mental and emotional health. In 2022, caregivers who ranked their mental and emotional health as fair or poor had 2.338 higher odds (*p* = 0.014, 1.191–4.591) and 4.872 higher odds (*p* < 0.001, 2.013–11.792) of having a child with severe behavioral or conduct problems compared to a caregiver with excellent mental and emotional health, respectively. All other associations were not significant (Table 2).

#### 3.2.3. ADD/ADHD

In 2019, caregivers who ranked their mental and emotional health as good or fair had 2.722 higher odds (*p* = 0.001, 1.535–4.829) and 4.109 higher odds (*p* < 0.001, 2.088–8.083) of having a child with severe ADD or ADHD compared to a caregiver with excellent mental and emotional health, respectively. In 2020, caregivers who ranked their mental and emotional health as fair had 2.654 higher odds (*p* < 0.001, 1.561–4.514) of having a child with severe ADD or ADHD compared to a caregiver with excellent mental and emotional health. In 2021, caregivers who ranked their mental and emotional health as fair had 2.625 higher odds (*p* < 0.001, 1.624–4.244) of having a child with severe ADD or ADHD compared to a caregiver with excellent mental and emotional health. In 2022, caregivers who ranked their mental and emotional health as good, fair, or poor had 2.269 higher odds (*p* = 0.001, 1.420–3.623), 4.553 higher odds (*p* < 0.001, 2.713–7.642), and 4.435 higher odds (*p* < 0.001, 1.986–9.908) of having a child with severe ADD or ADHD compared to a caregiver with excellent mental and emotional health, respectively. All other associations were not significant (Table 3).

#### 3.2.4. Depression and Autism/ASD

No significant associations were found for either depression (Table 4) or autism/ASD (Table 5).

## 4. Discussion

The present study seeks to address the existing gap in the literature by offering new estimates of changes in the national prevalence of childhood behavioral (behavioral conduct problems), mental health (anxiety and depression), and developmental disorders (ASD and ADD) among children ages 6–11 between 2019 and 2022. We examine both the overall burden of these childhood issues and caregiver mental and emotional well-being. Using data from the NSCH, we identified prominent links between childhood rates of behavioral, psychiatric, and developmental disorders and caregiver self-reports of mental and emotional health.

Our study found significant associations between severe childhood anxiety and “fair” or “poor” ratings of caregiver mental and emotional health for both the 2020 and 2022 survey years, illustrated by strikingly higher odds for these caregivers’ children having severe anxiety. These findings track with the results of several other recent studies [22,23,24,25], which report caregiver mental health having a direct and profound impact on the likelihood of a child developing anxiety. For instance, one study [22] found that children of a caregiver of either sex with poor mental health were more likely to have at least one mental, behavioral, or developmental disorder. This also relates to the recent rise in the incidence and prevalence of anxiety symptoms in children during and immediately following the COVID-19 pandemic, as this period was unique in the markedly increased amount of time children were spending with their caregivers [26,27,28]. As the association found in our study between severe childhood anxiety and lower caregiver rankings of mental and emotional health continued into 2022, a period in which COVID-19 mitigation measures began to relax, the psychological influence that caregivers have on children may not be transient but rather long-lasting and deeply ingrained. These findings support prospects of an increase in the need for developmental health services, thus necessitating efforts towards effective allocation and focus of developmental treatment programs.

In addition to our findings on childhood anxiety, our analysis also highlighted significant associations between caregiver mental and emotional health and the rate of child behavioral or conduct problems. For survey years 2019, 2020, and 2022, we found that caregiver rankings of “fair” or “poor” emotional and mental health corresponded to substantially higher odds for children displaying severe behavioral or conduct problems. This is particularly notable when compared to the associations we identified for severe childhood anxiety, as the association concerning behavioral or conduct problems was additionally significant in 2019. As the COVID-19 pandemic began in 2020, statistical significance in 2019 indicates that these associations are not an artificial product of the environment brought about by the COVID-19 mitigation measures but are rather derived from caregivers’ impact on childhood development. Multiple studies [29,30,31,32] conducted across a broad range of populations, from rural sub-Saharan Africa to the UK, support these findings. For example, a year-long study by [29] found that mothers who reported substantial depressive symptoms had children who exhibited significantly more behavior problems at age 4. Consequently, our findings indicate a greater need for access and enrollment in psychological health services for caregivers, as optimizing caregivers’ emotional and mental health could have downstream effects on minimizing child behavioral or conduct problems.

Our study also found that caregiver mental and emotional health may play a role in childhood ADD and ADHD prevalence rates as caregivers who ranked their mental health as “fair” or “good” had significantly higher odds of having a child with severe ADD or ADHD than caregivers who reported “excellent” mental and emotional health. These results are supported by previous findings [33] that children of mothers with activity-limiting depression, anxiety, or emotional problems were more than four times more likely to have ADHD, even after controlling for covariates like sociodemographic factors, household income, and family structure. Another study [34] identified a link between high parental psychological distress and dysfunctional parenting behaviors, including increased allowance of children’s screen media use, which in turn was significantly correlated with child ADHD-related symptoms. In a case–control study [35], it was found that parents of children with ADHD reported higher levels of ADHD and depressive symptoms than parents of children without ADHD. Several studies [36,37] have also suggested a bidirectional relationship between caregiver well-being and the severity and comorbidity rates of children’s ADHD presentation with higher parenting stress simultaneously contributing to and being exacerbated by ADHD symptoms in their children.

This study found no significant associations between caregiver mental and emotional health and either autism or depression in children. This is contrary to the existing literature [38,39,40] that reports parental depression, anxiety, and poor mental health to be significantly associated with early development of childhood emotional dysregulation and depressive symptoms. Specifically, maternal depression both before and after the child is born has been shown to be a significant predictor of childhood-onset depression compared to children of parents with no history of depression [41]. While there has been a deficit in the literature addressing the impact of caregiver mental and emotional health on ASD presentation, many studies [42,43,44] have examined the effect of ASD presentation on parental well-being and found that caregiver burden and psychological distress were significantly associated with the severity of ASD symptoms. It is also notable that depression and ADHD have far lower frequencies than the other developmental disorders examined in this study, which may play a role in the non-significant results found.

The findings of this study overall suggest a need to create a holistic approach to the treatment of childhood mental, behavioral, and developmental disorders. These solutions should involve the primary caregivers and consider the relevant social determinants of health, including caregivers’ mental and emotional well-being, for sustained impact on decreasing the burden of these problems. In a review conducted by [45], social-emotional learning programs targeting children aged 4–6 years old and their parents highlighted effective strategies to deal with childhood behavioral and mental health problems such as self-control, emotional understanding, building self-esteem, relationships, and interpersonal problem-solving skills. Further, parents were taught cognitive-behavioral strategies, emotional awareness, and emotional regulation to improve their parenting approach so that they can better support their children. However, these interventions failed to take into consideration the mental and emotional health of these caregivers and focused on short-term outcomes rather than longitudinal changes in desired child behavioral and mental problems. Hence, there is a need for a multi-level approach for effective intervention and sustained impact in this population group.

Wakschlag et al. [46] proposes the *Mental Health, Earlier* roadmap as the future of prevention of mental health disorders in place of our current model of treatment after diagnosis. The framework focuses on the early childhood period, including during pregnancy, and aims to identify atypical patterns of behavior and promote self-regulation within the pediatric healthcare system through interventions that can be scaled to the population level [46]. Likewise, studies have shown that caregivers of children with developmental disorders have higher levels of parenting stress, anxiety, and depression [22,30,47], and addressing the bidirectional relationship of caregiver well-being and childhood developmental disorders is imperative to ensure that children and families are given the proper tools to address these complexities [48]. Early family-centered interventions have been proposed to have a positive impact on the quality of childcare, especially considering the caregiver’s role as an intervention agent and gatekeeper, as well as the increased rates of mental health impairment experienced by caregivers of children with MBDDs [49]. Such prevention and intervention strategies should prioritize promoting the neurobiological mental health foundation of the family as a unit by supporting healthy family environments, caregiver-child relationships, and family routines, while simultaneously supporting the caregivers’ emotional and behavioral health through trauma-informed, multidisciplinary care. This should include proactive incorporation of integrated mental health and social services through multigenerational dyadic interventions such as contingency management strategies [50].

### Clinical Implications

Pediatric health providers should advocate for enhanced alignment and coordination of mental health services across family-serving sectors, including pediatric health care, public health services, early childcare and education, child welfare, and family support services [51]. This approach aligns with the full vision of a child’s “medical home,” introduced by the American Academy of Pediatrics in 1992, which calls for a family-centered partnership embedded in a community-based system that ensures access to uninterrupted care with appropriate payment to support and sustain optimal health outcomes [52]. Effective evidence-based interventions require cross-sector collaboration across health, social, and educational sectors rather than relying on addressing childhood mental health concerns solely in pediatric medical settings. For instance, programs such as Healthy Steps [53] and Help Me Grow [54], which were designed to improve community links and referrals for children experiencing developmental issues could be adapted to incorporate diverse aspects of mental health intervention for both children and parents instead of providing referrals to outside agencies or providers lacking practice-based communication strategies essential for sustained multidisciplinary care. However, the ability of pediatric health providers to create this type of integrated response to address childhood behavioral and mental health problems will be limited unless it is accompanied with a parallel attempt to redesign the entire early childhood health system to ensure expertise and capacity needed to mitigate the impact of identified issues early on to achieve optimal family health through a new vision for child health development [50,55,56].

## 5. Limitations

While our study is comprehensive, limitations do exist in some regards. For example, although NSCH data are broadly representative of the U.S. population, longitudinal rather than cross-sectional data are more accurate at assessing the change in trends in childhood behavioral, developmental, and mental problems across a specific timeframe. However, assembling large data via this national survey allows us to capture national populations and change in desired outcomes across multiple time points without non-random attrition. Recall bias is a notable concern, as parents may not accurately remember past events or details about their child’s behaviors and symptoms. This can lead to either underreporting or overreporting, thus distorting the true prevalence and nature of these conditions. Social desirability bias further complicates the reliability of parental reports. Parents may be inclined to present their children and themselves in a favorable light, consciously or unconsciously downplaying behavioral problems or exaggerating positive attributes. As a result, the data collected may not fully reflect the reality of the child’s experiences and challenges. Finally, we were unable to explore associations between specific factors measuring emotional and mental health of caregivers since only one question on this criterion was included in the survey. Future NSCH surveys should include specific caregiver mental and emotional health to further examine their impact on childhood mental, behavioral, and developmental health issues.

## 6. Conclusions

In conclusion, this study shows the need to address U.S. childhood behavioral, mental, and developmental problems in the 6–11 age group category, particularly when it comes to mitigating the impact of caregiver mental/emotional health on the exacerbation of such problems. Considering the importance of improving adolescent mental health to decrease the overall socioeconomic burden of childhood mental and developmental problems, evidence-based programs and treatments should be tailored to adopt a comprehensive approach, targeting not only the child, but also their caregivers and influential factors in their surrounding environment. Such prevention and intervention strategies should prioritize promoting the neurobiological mental health foundation of the family as a unit by supporting healthy family environments, caregiver–child relationships, and family routines, while simultaneously supporting the caregivers’ emotional and behavioral health through trauma-informed, multidisciplinary care. Moreover, considering the lingering impact of the COVID-19 pandemic on children mental health outcomes, it becomes a necessity for policymakers and public health professionals to take into consideration external factors and their dynamic impact on desired mental health outcomes when designing public health strategies and programs aimed at mitigating childhood behavioral and mental health problems.

## Figures and Tables

**Table 1:** Binary Logistic Regression of Childhood Anxiety with Caregiver Mental/Emotional Health Rating.

Independent Variable (Caregiver Mental and Emotional Health)	Anxiety																			
	2019					2020					2021					2022				
	Non-Severe	Severe	OR	95%	*p*-Value	Non-Severe	Severe	OR	95%	*p*-Value	Non-Severe	Severe	OR	95%	*p*-Value	Non-Severe	Severe	OR	95%	*p*-Value
Excellent	129 (16%)	6 (10.5%)			0.245	163 (14.8%)	14 (14.6%)			0.001	135 (10.8%)	15 (14.2%)			0.025	174 (11.4%)	9 (7.1%)			<0.001
Very Good	324 (40.1%)	18 (31.6%)	1.194	0.464–3.077	0.713	399 (36.3%)	22 (22.9%)	0.642	0.321–1.286	0.211	449 (35.8%)	30 (28.3%)	0.601	0.314–1.151	0.125	543 (35.7%)	39 (30.1%)	1.389	0.659–2.924	0.388
Good	264 (32.7%)	22 (38.6%)	1.792	0.709–4.527	0.218	380 (34.5%)	30 (31.3%)	0.919	0.475–1.779	0.802	460 (36.7%)	33 (31.1%)	0.646	0.341–1.224	0.180	553 (36.3%)	40 (31.7%)	1.398	0.665–2.940	0.376
Fair	78 (9.7%)	9 (15.8%)	2.481	0.850–7.236	0.096	136 (12.3%)	27 (28.1%)	2.311 *	1.166–4.583	0.016	183 (14.6%)	27 (25.5%)	1.328	0.680–2.593	0.406	217 (14.2%)	27 (21.4%)	2.406 *	1.102–5.249	0.027
Poor	13 (1.6%)	2 (3.5%)	3.308	0.605–18.086	0.168	25 (2.3%)	3 (3.1%)	1.397	0.375–5.210	0.618	27 (2.2%)	1 (0.9%)	0.333	0.042–2.631	0.297	36 (2.4%)	11 (8.7%)	5.907 *	2.282–15.294	<0.001

* *p* < 0.05.

**Table 2:** Binary Logistic Regression of Childhood Behavioral or Conduct Problems with Caregiver Mental/Emotional Health Rating.

Independent Variable (Caregiver Mental and Emotional Health)	Behavior or Conduct Problems																			
	2019					2020					2021					2022				
	Non-Severe	Severe	OR	95%	*p*-Value	Non-Severe	Severe	OR	95%	*p*-Value	Non-Severe	Severe	OR	95%	*p*-Value	Non-Severe	Severe	OR	95%	*p*-Value
Excellent	144 (20.1%)	10 (12.7%)			0.134	179 (15.8%)	16 (13.3%)			0.026	190 (15.6%)	15 (12.1%)			0.200	190 (13.6%)	13 (9.7%)			0.000
Very Good	263 (36.7%)	23 (29.1%)	1.259	0.583–2.719	0.557	425 (37.6%)	38 (31.7%)	1.000	0.544–1.840	0.999	419 (34.3%)	35 (28.2%)	1.058	0.564–1.984	0.860	471 (33.6%)	28 (20.9%)	0.869	0.441–1.713	0.685
Good	208 (29.1%)	29 (36.7%)	2.008	0.949–4.248	0.068	353 (31.2%)	35 (29.2%)	1.109	0.598–2.058	0.742	408 (33.4%)	44 (35.5%)	1.366	0.742–2.516	0.317	506 (36.1%)	50 (37.3%)	1.444	0.767–2.719	0.255
Fair	88 (12.3%)	15 (19%)	2.455 *	1.057–5.702	0.037	156 (13.8%)	25 (20.8%)	1.793	0.924–3.480	0.084	179 (14.7%)	27 (21.8%)	1.911	0.984–3.709	0.056	200 (14.3%)	32 (23.9%)	2.338 *	1.191–4.591	0.014
Poor	13 (1.8%)	2 (2.5%)	2.215	0.438–11.204	0.336	18 (1.6%)	6 (5%)	3.729 *	1.297–10.721	0.015	24 (2%)	3 (2.4%)	1.583	0.427–5.870	0.492	33 (2.4%)	11 (8.2%)	4.872 *	2.013–11.792	<0.001

* *p* < 0.05.

**Table 3:** Binary Logistic Regression of Childhood ADD/ADHD with Caregiver Mental/Emotional Health Rating.

Independent Variable (Caregiver Mental and Emotional Health)	ADD/ADHD																			
	2019					2020					2021					2022				
	Non-Severe	Severe	OR	95%	*p*-Value	Non-Severe	Severe	OR	95%	*p*-Value	Non-Severe	Severe	OR	95%	*p*-Value	Non-Severe	Severe	OR	95%	*p*-Value
Excellent	209 (25.4%)	18 (13.1%)			0.000	261 (21.6%)	30 (15.5%)			0.002	236 (18%)	35 (16.1%)			0.000	300 (18.4%)	24 (9.5%)			0.000
Very Good	329 (40%)	44 (32.19%)	1.553	0.874–2.760	0.134	477 (39.5%)	65 (33.7%)	1.186	0.750–1.875	0.467	509 (38.9%)	62 (28.4%)	0.821	0.528–1.278	0.383	607 (37.3%)	65 (25.7%)	1.339	0.822–2.181	0.242
Good	209 (25.4%)	49 (35.8%)	2.722 *	1.535–4.829	0.001	336 (27.8%)	58 (30.1%)	1.502	0.939–2.402	0.090	419 (32%)	68 (31.2%)	1.094	0.706–1.695	0.687	540 (33.1%)	98 (38.7%)	2.269 *	1.420–3.623	0.001
Fair	65 (7.9%)	23 (16.8%)	4.109 *	2.088–8.083	<0.001	118 (9.8%)	36 (18.7%)	2.654 *	1.561–4.514	<0.001	131 (10%)	51 (23.4%)	2.625 *	1.624–4.244	0.000	151 (9.3%)	55 (21.7%)	4.553 *	2.713–7.642	<0.001
Poor	11 (1.3%)	3 (2.2%)	3.167	0.809–12.392	0.098	16 (1.3%)	4 (2.1%)	2.175	0.683–6.931	0.189	13 (1%)	2 (0.9%)	1.037	0.225–4.793	0.963	31 (1.9%)	11 (4.3%)	4.435 *	1.986–9.908	<0.001

* *p* < 0.05.

**Table 4:** Binary Logistic Regression of Childhood Depression with Caregiver Mental/Emotional Health Rating.

Independent Variable (Caregiver Mental and Emotional Health)	Depression																			
	2019					2020					2021					2022				
	Non-Severe	Severe	OR	95%	*p*-Value	Non-Severe	Severe	OR	95%	*p*-Value	Non-Severe	Severe	OR	95%	*p*-Value	Non-Severe	Severe	OR	95%	*p*-Value
Excellent	21 (11%)	2 (18.2%)			0.759	27 (11%)	3 (21.4%)			0.821	23 (8.7%)	1 (5.9%)			0.402	26 (8%)	1 (4.5%)			0.154
Very Good	51 (26.7%)	1 (9.1%)	0.206	0.018–2.390	0.207	63 (25.7%)	3 (21.4%)	0.429	0.081–2.260	0.318	80 (30.3%)	2 (11.8%)	0.575	0.050–6.629	0.657	86 (26.3%)	1 (4.5%)	0.302	0.018–5.003	0.403
Good	75 (39.3%)	5 (45.5%)	0.700	0.127–3.870	0.683	88 (35.9%)	4 (28.6%)	0.409	0.086–1.943	0.261	105 (39.8%)	8 (47.1%)	1.752	0.209–14.706	0.605	136 (41.6%)	12 (54.6%)	2.294	0.286–18.413	0.435
Fair	35 (18.3%)	3 (27.3%)	0.900	0.139–5.835	0.912	58 (23.7%)	4 (28.6%)	0.621	0.130–2.969	0.550	48 (18.2%)	6 (35.3%)	2.875	0.327–25.295	0.341	65 (19.9%)	5 (22.7%)	2.000	0.223–17.954	0.536
Poor	9 (4.7%)	0 (0%)	0	0–0	0.999	9 (3.7%)	0 (0%)	0	0–0	0.999	8 (3%)	0 (0%)	0	0–0	0.999	14 (4.3%)	3 (13.6%)	5.571	0.529–58.688	0.153

**Table 5:** Binary Logistic Regression of Childhood ASD with Caregiver Mental/Emotional Health Rating.

Independent Variable (Caregiver Mental and Emotional Health)	Autism Spectrum Disorder																			
	2019					2020					2021					2022				
	Non-Severe	Severe	OR	95%	*p*-Value	Non-Severe	Severe	OR	95%	*p*-Value	Non-Severe	Severe	OR	95%	*p*-Value	Non-Severe	Severe	OR	95%	*p*-Value
Excellent	47 (19.1%)	5 (18.5%)			0.050	70 (19.7%)	7 (14.9%)			0.726	74 (19.4%)	15 (22.7%)			0.557	103 (19.1%)	11 (15.3%)			0.831
Very Good	101 (41.1%)	6 (22.2%)	0.558	0.162–1.922	0.356	127 (35.8%)	17 (36.2%)	1.339	0.530–3.383	0.538	137 (35.9%)	17 (25.8%)	0.612	0.289–1.296	0.199	181 (33.6%)	28 (38.9%)	1.449	0.692–3.031	0.325
Good	78 (31.7%)	9 (33.3%)	1.085	0.343–3.431	0.890	108 (30.4%)	13 (27.7%)	1.204	0.458–3.165	0.707	113 (29.6%)	21 (31.8%)	0.917	0.444–1.892	0.814	172 (31.9%)	22 (30.6%)	1.198	0.558–2.571	0.643
Fair	17 (6.9%)	5 (18.5%)	2.765	0.711–10.751	0.142	44 (12.4%)	9 (19.5%)	2.045	0.711–5.888	0.185	51 (13.4%)	12 (18.3%)	1.161	0.502–2.685	0.728	69 (12.8%)	10 (13.9%)	1.357	0.547–3.368	0.510
Poor	3 (1.2%)	2 (7.4%)	6.267	0.837–46.9	0.074	6 (1.7%)	1 (2.1%)	1.667	0.175–15.893	0.657	7 (1.8%)	1 (1.5%)	0.705	0.081–6.157	0.752	14 (2.6%)	1 (1.4%)	0.669	0.08–5.583	0.710

## Data Availability

The authors used data from a national public dataset, the National Survey of Children’s Health, and can share the specific datasets used upon request.

## References

[1] Scott J.G., Mihalopoulos C., Erskine H.E., Roberts J., Rahman A., Patel V., Chisholm D., Dua T., Laxminarayan R., Medina-Mora M.E. (2016). Childhood Mental and Developmental Disorders. Mental, Neurological, and Substance Use Disorders: Disease Control Priorities.

[2] Glassgow A.E., Wilder J., Caskey R., Munoz G., Van Voorhees B., Kim S. (2020). Mental Health Diagnoses among Children and Adolescents with Chronic Medical Conditions in a Large Urban Cohort. J. Behav. Health.

[3] Ogundele M.O. (2018). Behavioural and emotional disorders in childhood: A brief overview for paediatricians. World J. Clin. Pediatr..

[4] Child and Adolescent Mental Health (2022). 2022 National Healthcare Quality and Disparities Report [Internet].

[5] Tkacz J., Brady B.L. (2021). Increasing rate of diagnosed childhood mental illness in the United States: Incidence, prevalence and costs. Public Health Pract..

[6] Cree R.A., Bitsko R.H., Robinson L.R., Holbrook J.R., Danielson M.L., Smith C., Kaminski J.W., Kenney M.K., Peacock G. (2018). Health Care, Family, and Community Factors Associated with Mental, Behavioral, and Developmental Disorders and Poverty Among Children Aged 2–8 Years—United States, 2016. Morb. Mortal. Wkly. Rep..

[7] Leeb R.T., Bitsko R.H., Radhakrishnan L., Martinez P., Njai R., Holland K.M. (2020). Mental Health–Related Emergency Department Visits Among Children Aged < 18 Years During the COVID-19 Pandemic—United States, January 1–October 17, 2020. Morb. Mortal. Wkly. Rep..

[8] Juwariah T., Suhariadi F., Soedirham O., Priyanto A., Setiyorini E., Siskaningrum A., Adhianata H., Fernandes A.d.C. (2022). Childhood adversities and mental health problems: A systematic review. J. Public Health Res..

[9] Costello E.J. (2016). Early Detection and Prevention of Mental Health Problems: Developmental Epidemiology and Systems of Support. J. Clin. Child Adolesc. Psychol..

[10] Bornstein M.H. (2013). Parenting and child mental health: A cross-cultural perspective. World Psychiatry.

[11] Morrison Gutman L., McLoyd V.C., Tokoyawa T. (2005). Financial Strain, Neighborhood Stress, Parenting Behaviors, and Adolescent Adjustment in Urban African American Families. J. Res. Adolesc..

[12] Parfitt Y., Pike A., Ayers S. (2013). The impact of parents’ mental health on parent-baby interaction: A prospective study. Infant Behav. Dev..

[13] Quinn A., Briggs H.E., Miller K.M., Orellana E.R. (2014). Social and familial determinants of health: Mediating effects of caregiver mental and physical health on children’s mental health. Child. Youth Serv. Rev..

[14] Mensah F.K., Kiernan K.E. (2010). Parents’ mental health and children’s cognitive and social development: Families in England in the Millennium Cohort Study. Soc. Psychiatry Psychiatr. Epidemiol..

[15] Likhar A., Baghel P., Patil M. (2022). Early Childhood Development and Social Determinants. Cureus.

[16] Bureau U.C. National Survey of Children’s Health (NSCH). https://www.census.gov/nsch.

[17] National Survey of Children’s Health—Data Resource Center for Child and Adolescent Health. https://www.childhealthdata.org/learn-about-the-nsch/NSCH.

[18] Wolf K., Schmitz J. (2024). Scoping review: Longitudinal effects of the COVID-19 pandemic on child and adolescent mental health. Eur. Child Adolesc. Psychiatry.

[19] Amrock S.M., Weitzman M. (2014). Parental Psychological Distress and Children’s Mental Health: Results of a National Survey. Acad. Pediatr..

[20] Dayton L., Kong X., Powell T.W., Bowie J., Rebok G., Strickland J.C., Latkin C. (2022). Child mental health and sleep disturbances during the early months of the COVID-19 pandemic in the United States. Fam. Community Health.

[21] van Santvoort F., Hosman C.M.H., Janssens J.M.A.M., van Doesum K.T.M., Reupert A., van Loon L.M.A. (2015). The Impact of Various Parental Mental Disorders on Children’s Diagnoses: A Systematic Review. Clin. Child Fam. Psychol. Rev..

[22] Wolicki S.B., Bitsko R.H., Cree R.A., Danielson M.L., Ko J.Y., Warner L., Robinson L.R. (2021). Mental Health of Parents and Primary Caregivers by Sex and Associated Child Health Indicators. Advers. Resil. Sci..

[23] Leijdesdorff S., van Doesum K., Popma A., Klaassen R., van Amelsvoort T. (2017). Prevalence of psychopathology in children of parents with mental illness and/or addiction: An up to date narrative review. Curr. Opin. Psychiatry.

[24] Wickersham A., Leightley D., Archer M., Fear N.T. (2020). The association between paternal psychopathology and adolescent depression and anxiety: A systematic review. J. Adolesc..

[25] Chapman L., Hutson R., Dunn A., Brown M., Savill E., Cartwright-Hatton S. (2022). The impact of treating parental anxiety on children’s mental health: An empty systematic review. J. Anxiety Disord..

[26] Hawes M.T., Szenczy A.K., Klein D.N., Hajcak G., Nelson B.D. (2022). Increases in depression and anxiety symptoms in adolescents and young adults during the COVID-19 pandemic. Psychol. Med..

[27] Madigan S., Racine N., Vaillancourt T., Korczak D.J., Hewitt J.M.A., Pador P., Park J.L., McArthur B.A., Holy C., Neville R.D. (2023). Changes in Depression and Anxiety Among Children and Adolescents From Before to During the COVID-19 Pandemic. JAMA Pediatr..

[28] Ben Brik A., Williams N., Esteinou R., Acero I.D.M., Mesurado B., Debeliuh P., Storopoli J.E., Orellana O.N., James S.L. (2022). Parental mental health and child anxiety during the COVID-19 pandemic in Latin America. J. Soc. Issues.

[29] Hoffman C., Crnic K.A., Baker J.K. (2006). Maternal Depression and Parenting: Implications for Children’s Emergent Emotion Regulation and Behavioral Functioning. Parenting.

[30] Laurenzi C.A., Hunt X., Skeen S., Sundin P., Weiss R.E., Kosi V., Rotheram-Borus M.J., Tomlinson M. (2021). Associations between caregiver mental health and young children’s behaviour in a rural Kenyan sample. Glob. Health Action.

[31] Fitzsimons E., Goodman A., Kelly E., Smith J.P. (2017). Poverty dynamics and parental mental health: Determinants of childhood mental health in the UK. Soc. Sci. Med. 1982.

[32] Elgar F.J., McGrath P.J., Waschbusch D.A., Stewart S.H., Curtis L.J. (2004). Mutual influences on maternal depression and child adjustment problems. Clin. Psychol. Rev..

[33] Lesesne C.A., Visser S.N., White C.P. (2003). Attention-deficit/hyperactivity disorder in school-aged children: Association with maternal mental health and use of health care resources. Pediatrics.

[34] Waller F., Prandstetter K., Jansen E., Nikolova G., Lachman J.M., Hutchings J., Foran H.M. (2023). Screen use: Its association with caregiver mental health, parenting, and children’s ADHD symptoms. Fam. Relat..

[35] Margari F., Craig F., Petruzzelli M.G., Lamanna A., Matera E., Margari L. (2013). Parents psychopathology of children with Attention Deficit Hyperactivity Disorder. Res. Dev. Disabil..

[36] Rockhill C., Violette H., Stoep A.V., Grover S., Myers K. (2013). Caregivers’ Distress: Youth with Attention-Deficit/Hyperactivity Disorder and Comorbid Disorders Assessed via Telemental Health. J. Child Adolesc. Psychopharmacol..

[37] Segenreich D., Fortes D., Coutinho G., Pastura G., Mattos P. (2009). Anxiety and depression in parents of a Brazilian non-clinical sample of attention-deficit/ hyperactivity disorder (ADHD) students. Braz. J. Med. Biol. Res..

[38] England M.J., Sim L.J., National Research Council (US) and Institute of Medicine (US) Committee on Depression, Parenting Practices, and the Healthy Development of Children (2009). Depression in Parents, Parenting, and Children: Opportunities to Improve Identification, Treatment, and Prevention.

[39] Meyer S.R., Steinhaus M., Bangirana C., Onyango-Mangen P., Stark L. (2017). The influence of caregiver depression on adolescent mental health outcomes: Findings from refugee settlements in Uganda. BMC Psychiatry.

[40] Moffitt T.E., Caspi A., Harrington H., Milne B.J., Melchior M., Goldberg D., Poulton R. (2007). Generalized anxiety disorder and depression: Childhood risk factors in a birth cohort followed to age 32. Psychol. Med..

[41] Brophy S., Todd C., Rahman M.A., Kennedy N., Rice F. (2021). Timing of parental depression on risk of child depression and poor educational outcomes: A population based routine data cohort study from Born in Wales, UK. PLoS ONE.

[42] Baykal S., Karakurt M.N., Çakır M., Karabekiroğlu K. (2019). An Examination of the Relations Between Symptom Distributions in Children Diagnosed with Autism and Caregiver Burden, Anxiety and Depression Levels. Community Ment. Health J..

[43] Herrero R., Díaz A., Zueco J. (2024). The Burden and Psychological Distress of Family Caregivers of Individuals with Autism Spectrum Disorder: A Gender Approach. J. Clin. Med..

[44] Hickey E.J., Hartley S.L., Papp L. (2020). Psychological Well-Being and Parent-Child Relationship Quality in Relation to Child Autism: An Actor-Partner Modeling Approach. Fam. Process.

[45] Baughman N., Prescott S.L., Rooney R. (2020). The Prevention of Anxiety and Depression in Early Childhood. Front.Psychol..

[46] Wakschlag L.S., Roberts M.Y., Flynn R.M., Smith J.D., Krogh-Jespersen S., Kaat A.J., Gray L., Walkup J., Marino B.S., Norton E.S. (2019). Future Directions for Early Childhood Prevention of Mental Disorders: A Roadmap to Mental Health, Earlier. J. Clin. Child Adolesc. Psychol..

[47] Wang C. (2021). Mental health and social support of caregivers of children and adolescents with ASD and other developmental disorders during COVID-19 pandemic. J. Affect. Disord. Rep..

[48] CDC Mental Health of Children and Parents—A Strong Connection. https://www.cdc.gov/children-mental-health/about/index.html.

[49] Bagur S., Paz-Lourido B., Mut-Amengual B., Verger S. (2022). Relationship between parental mental health and developmental disorders in early childhood. Health Soc. Care Community.

[50] Buka S.L., Beers L.S., Biel M.G., Counts N.Z., Hudziak J., Parade S.H., Paris R., Seifer R., Drury S.S. (2022). The Family is the Patient: Promoting Early Childhood Mental Health in Pediatric Care. Pediatrics.

[51] Willis D. Advancing a Family Centered Community Health System: A community Agenda Focused on Child Health Care, Early Relationships and Equity. https://earlychildhoodimpact.org/resource/advancing-a-family-centered-community-health-system-a-community-agenda-focused-on-child-health-care-foundational-relationships-and-equity/.

[52] American Academy of Pediatrics AAP Agenda for Children: Medical Home. https://www.aap.org/en/practice-management/medical-home/?srsltid=AfmBOoqkz_1WBtWDhyfkelbi64ZYBhT260EHgB0ervR3D61Kpp5MmvTi.

[53] Piotrowski C.C., Talavera G.A., Mayer J.A. (2009). Healthy steps: A systematic review of a preventive practice-based model of pediatric care. J. Dev. Behav. Pediatr..

[54] Bogin J. (2006). Enhancing developmental services in primary care: The Help Me Grow experience. J. Dev. Behav. Pediatr..

[55] Alsaad A.J., Azhar Y., Al Nasser Y. (2004). Depression in Children. StatPearls.

[56] CDC Data and Statistics on Children’s Mental Health. https://www.cdc.gov/children-mental-health/data-research/index.html.

